# Neural mechanisms underlying synchronization of movement to musical cues in Parkinson disease and aging

**DOI:** 10.3389/fnins.2025.1550802

**Published:** 2025-03-11

**Authors:** Elinor C. Harrison, Sarah Grossen, Lauren E. Tueth, Allison M. Haussler, Kerri S. Rawson, Meghan C. Campbell, Gammon M. Earhart

**Affiliations:** ^1^Program in Physical Therapy, Washington University in St. Louis School of Medicine, St. Louis, MO, United States; ^2^Performing Arts Department, Washington University in St. Louis, St. Louis, MO, United States; ^3^Department of Neurology, Washington University in St. Louis School of Medicine, St. Louis, MO, United States; ^4^Department of Radiology, Washington University in St. Louis School of Medicine, St. Louis, MO, United States; ^5^Department of Neuroscience, Washington University in St. Louis School of Medicine, St. Louis, MO, United States

**Keywords:** music, mental singing, rhythm, gait rehabilitation, Parkinson disease

## Abstract

**Introduction:**

External and internal musical cues provide therapeutic techniques for gait rehabilitation in aging and neurological disorders. For people with Parkinson disease (PwPD), mental singing is a type of internal cue that can regularize gait timing. No studies to date have directly measured brain activity during external and internal musical cues as used in gait rehabilitation. Evidence suggests the neural mechanisms of external vs. internal cued movement differ. External cues are thought to drive movement via recruitment of cerebello-thalamo-cortical (CTC) pathways, while internal cues are thought to rely more on striato-pallido-thalamocortical (SPT) pathways.

**Methods:**

We investigated the neural mechanisms that underlie acute responses to external cues (listening to music) and internal cues (mental singing). Using fMRI, we imaged PwPD and age-matched healthy controls (HC) while performing finger tapping during musical cueing tasks.

**Results:**

No differences were seen between PwPD and HC in any of the comparisons. Functional imaging results showed activation of sensorimotor cortex, temporal gyri, supplementary motor areas, and putamen for both cueing tasks. External cues additionally activated auditory cortex while internal cues additionally activated the cerebellum. When directly comparing cue types, external cues displayed greater activity in the primary auditory cortex and temporal gyri.

**Discussion:**

These results suggest similar brain regions are activated during musically-cued movements for both PwPD and HC and both cue types utilize parallel pathways for processing. Both cue types may facilitate use of remaining function of areas that degenerate in PD (e.g., putamen) and potentially also activate routes through less impaired areas (e.g., cerebellum). This supports the idea that the CTC and SPT pathways work in tandem and facilitate sensorimotor activity via a complex interplay between neural circuits. These findings have implications for how external and internal cues may be administered in future therapies.

## 1 Introduction

Walking is an inherently rhythmic activity that relies on stable timing mechanisms in the brain. For people with Parkinson disease (PwPD), basal ganglia degeneration may cause faulty internal timing that can destabilize gait. External rhythmic auditory stimulation (RAS) in the form of cues provide a source of regular input that may facilitate movement timing through auditory-motor coupling (Thaut et al., 2014). Synchronizing movement to the beat of the external rhythm may create a template for anticipation of the timing of future events (Nombela, 2013).

Internal rhythmic cueing, such as mental singing, is a technique that holds promise for gait rehabilitation as it is readily accessible, low-cost, and easily translatable into home and community settings (Harrison et al., 2017). Evidence suggests mental singing is neither rare nor difficult, as people report easily singing songs in their heads or getting stuck with earworms (Thaut et al., 2014).

Work from our lab shows mental singing is as effective as external cueing at improving gait speed and may be even more effective at improving gait stability for both older adults and PwPD (Harrison et al., 2018). Gait timing tends to regularize during mental singing tasks, suggesting that internal cues allow for greater stability of timing mechanisms (Harrison et al., 2019). We showed similar results during finger tapping tasks, suggesting that finger tapping can be used as a proxy for gait (Horin et al., 2021). While our recent work suggests key differences in the effects of external musical cues vs. self-generated singing cues on movement performance in PwPD and healthy controls (HC) (Harrison et al., 2018; Harrison et al., 2017), no studies to date have directly measured and compared brain activity during these different cueing conditions.

Auditory-motor coupling in the brain is well-documented. Brain areas involved in rhythm processing overlap substantially with those involved in motor control (e.g., premotor and supplementary motor cortices, cerebellum, basal ganglia) (Bengtsson et al., 2009; Chen et al., 2008; Grahn and Brett, 2007). In fact, coupling between auditory and premotor cortices is enhanced during rhythm processing (Chen et al., 2006; Grahn and Rowe, 2009). The putamen, in particular, is implicated in rhythmic event sequencing and beat perception (Grahn and Rowe, 2009; McIntosh, 1997). The cerebellum may also play a role in monitoring ongoing rhythmic movements and adjusting to changing tempos (Bijsterbosch et al., 2011; Thaut et al., 2009).

For individuals with PD, activity in the putamen, cerebral cortex, and corticostriatal pathways is reduced during uncued movements (Disbrow et al., 2013). Previous reports show restored activity of these areas with the application of external cues that is more similar to that of control participants (Taniwaki et al., 2013). Thus, external cues are thought to facilitate sensorimotor network activity and executive/attentional control as evidenced by increased prefrontal cortex activity when walking with cues (Vitorio et al., 2018).

**Figure 1:**
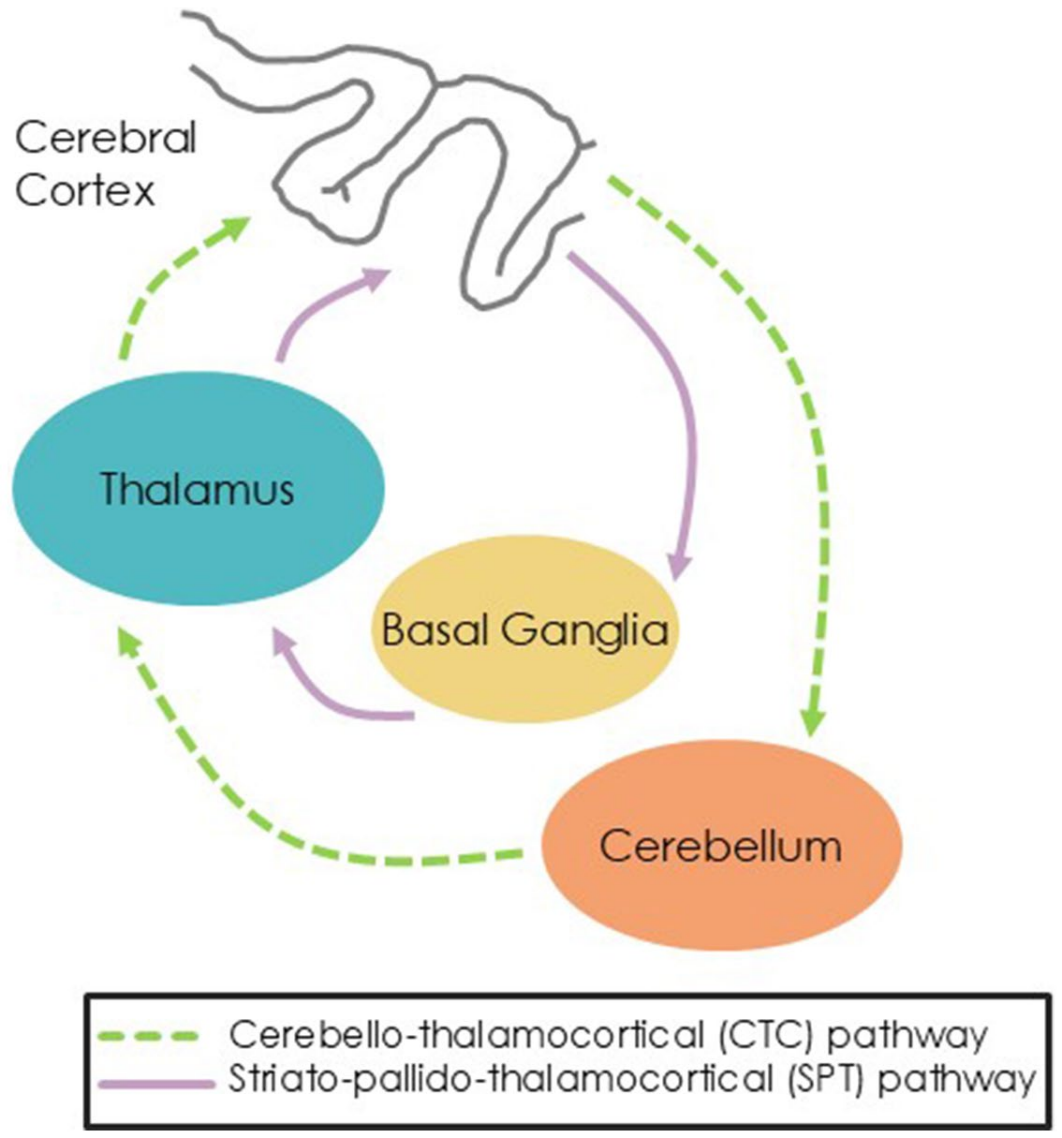
Hypothesized pathways showing external cues routing through the CTC pathway and internal cues routing through the SPT pathway.

Whereas external cues are thought to drive movement via recruitment of cerebello-thalamo-cortical (CTC) pathways, internal cues are thought to rely more on striato-pallido-thalamocortical (SPT) pathways (Sen et al., 2010). Dysfunction of the SPT circuit due to dopamine depletion in the substantia nigra in PD suggests PwPD could have more difficulty with internally-cued movements than HC (Braunlich et al., 2019). However, PwPD should perform similarly to HC during externally-cued movements which are thought to rely on spared CTC pathways (Figure 1).

The primary purpose of this study was to investigate the brain mechanisms underlying acute responses to external cues (via listening to music) and internal cues (via mental singing) in PwPD and older adults. Our hypotheses were: (1) External cues will elicit greater activation of the cerebellar-thalamo-cortical (CTC) pathway whereas internal cues will elicit greater activation of the striato-pallado-thalamocortical (SPT) pathway for both PwPD and HC; and (2) as internal cueing presumably activates the SPT pathway, we expect PwPD will have less activation of the SPT due to striatal degeneration and more activation of the CTC pathway as compensation compared to HC.

## 2 Materials and methods

### 2.1 Participants

Participants were recruited from a larger study exploring the effects of external and internal cueing techniques on gait parameters. PwPD were recruited through the Movement Disorders Clinic at Washington University in St. Louis School of Medicine and through flyers given to the American Parkinson Disease Association. Age-and gender-matched controls were recruited through the Washington University Older Adults Participant Pool and the Research Participant Registry. Additional recruitment occurred through advertisements, flyers, and social media.

Inclusion criteria were as follows: (1) at least 30 years of age; (2) right-handed (criterion for MRI); (3) normal hearing; (4) able to walk for 10 continuous minutes independently; and (5) willing and able to provide informed consent. Additional inclusion criteria for PwPD included: (1) diagnosis of idiopathic, typical Parkinson disease according to the United Kingdom Brain Bank Criteria; (2) Hoehn & Yahr stages 2–3 (mild to moderate disease severity) (Hoehn & Yahr, 1967); (3) score of ≥1 on the Movement Disorders Society Unified Parkinson Disease Rating Scale – Part III – Motor Aspects (MDS-UPDRS-III) (Goetz et al., 2008) item #10, indicating observable gait impairment; (4) a score of 1 or less on item #7 on the New Freezing of Gait Questionnaire (nFOG-Q) (Nieuwboer et al., 2009), indicating freezing episodes are not moderately or significantly disturbing to daily walking; and (5) stable on all PD medications for at least 2 months prior to study entry. Potential participants were excluded if they had a diagnosis of any other neurological condition or other medical conditions that might interfere with safe participation or if they demonstrated significant cognitive impairment (i.e., Mini-Mental Status Examination (MMSE) score of <24).

### 2.2 Protocol

All participants came for two sessions, which occurred within 10 days of each other. For both sessions, PwPD were tested while on their normal PD medications to represent everyday conditions. Participants were asked if they felt their medication was working as normal for them and if they were in the “ON” state in their medication cycle prior to testing.

The first session involved behavioral and motor testing. Upon providing written informed consent, eligibility was determined during a pre-assessment that included a demographics questionnaire, a pure-tone electronic hearing test, the MMSE, the MDS-UPDRS-III (Goetz et al., 2008), and nFOG-Q (Nieuwboer et al., 2009). Eligible participants then proceeded with the first session, lasting 2–3 h.

#### 2.2.1 Personalized cue identification

The purpose of the first session was to identify each individual’s personalized cue tempo in order to tailor cues to individuals. Detailed information regarding the protocol for cue personalization is available (Harrison et al., 2025).

Briefly, cues were personalized based on song choice and cue rate. Each participant was allowed to choose their own song for cueing from a curated catalog of songs with features suitable for gait training such as 4/4 timing, beat salience, familiarity, and simple lyrics (Leman et al., 2013). Participants were asked to select the song most familiar to them as improvements in velocity and stride length have been seen in PwPD when synchronizing to a highly familiar song (de Bruin et al., 2010) and lyrics sung on a life-long familiar melody result in better consolidation and higher retention (Moussard et al., 2014).

Personalized cue rate was determined according to which tempo elicited the longest strides during gait assessment. Gait performance was measured using the Mobility Lab System gait and posture analysis system (APDM, Inc., Portland, OR) (Morris et al., 2019). Participants were instructed to perform gait tasks in two conditions: Music (external cue: participants listen to the music cue and walk to the beat) and Mental (internal cue: participants listen to the song one time through, then mentally sing the cue and walk to the beat). Both conditions were done at four individualized cue rates: 90, 100, 110, and 120% of preferred walking cadence. The range of cue tempos was chosen to reflect those most likely to improve gait for PwPD (Ghai et al., 2018).

Upon completion of all walking trials, stride lengths were averaged across the music and mental trials within each cue rate, and the cue rate that elicited the longest stride lengths during gait assessment was selected for personalization.

#### 2.2.2 Tapping assessment

After cue personalization, participants performed tapping tasks while seated at a computer. Uncued tapping was tested first. Their selected song was then adapted to match the percentage of uncued tapping tempo as determined from the gait assessment. For example, if a participant was personalized to 110% of preferred walking cadence, the cue rate for tapping would be set to 110% of the uncued tapping cadence. Participants then performed blocks of right-handed tapping during external cues (listening to music) and internal cues (mental singing) at their personalized tapping rate. The blocks were presented in random order and included five 30-s trials per block. Personalized cues identified during this session were used for the tapping tasks during the fMRI scans in the second session.

#### 2.2.3 MRI acquisition

Within 10 days, participants returned for a second visit to undergo task-based fMRI assessments.

Magnetic resonance images were acquired with a 3 T Siemens Prisma scanner using a 20-channel head coil. Each participant completed one scan session, lasting about 1.5 h. Two anatomical images were acquired: T1-weighted (TR = 2,400 ms, TI = 1,000 ms, TE = 3.18 ms, FA = 8°, TA = 8:09, 0.9 mm voxels) and T2-weighted (TR = 3,200 ms, TE = 294 ms, FA = 120°, TA = 5:38, 0.9 mm voxels).

Functional images were collected using an echo-planar imaging sequence (TR = 1,200 ms, TE = 32.4 ms, FA = 63°, TA = 6:38, 2.4 mm voxels, multiband factor of 4), one for each of five task conditions outlined below. A midsagittal scout T1-weighted pulse sequence (TR = 3.15 ms, TE = 1.37 ms, FA = 8°, TA = 0:14, 1.6 mm voxels) was used for positioning and GRE field map images (TR = 747 ms, TE1 = 4.92 ms, TE2 = 7.38 ms, FA = 60°, TA = 2:17, 2.4 mm voxels) were collected to correct for field inhomogeneities.

#### 2.2.4 Task design

**Figure 2:**
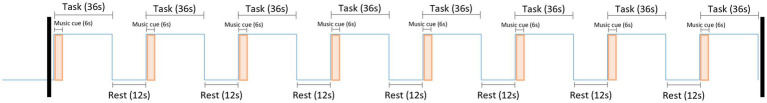
Block design used for fMRI task conditions. Bold black lines indicate the beginning and end of the scan sequence. Before the beginning of the sequence, the participant receives instructions. The sequence then begins with a task block lasting 36 s, followed by a 12 s rest. For the first 6 s of the task block for conditions involving mentally singing, the music is played as a cue.

Block designed task-based sequences included five conditions: (1) uncued tapping (Tap); (2) listening to music while tapping to the beat (Music+Tap); (3) mentally singing while tapping to the beat (Mental+Tap); (4) listening to music only (Music); and (5) mentally singing only (Mental). For each condition, the participant was given instructions at the start of the sequence, delivered auditorily through headphones and visually on a screen. Following instructions, a 36-s task block began, followed by a 12-s rest block, repeated seven times. For internally-cued conditions involving mentally singing (Mental and Mental+Tap), music was played during the first 6 sec of the task block to cue the melody to be mentally sung (Figure 2). For externally-cued conditions involving listening to music (Music and Music+Tap), the music played repeatedly during the task block and participants were reminded to not mentally sing.

The uncued tapping condition was completed first, followed by the others in random order. For the duration of the scan session, participants wore noise canceling headphones to minimize auditory impact of scanner noise. Participants tapped on a button box with their right index finger to the beat of the music.

Successful completion of the tapping tasks was determined via comparing average inter-tap-interval of tapping conditions. We calculated each participant’s percent ratio of Mental+Tap vs. Music+Tap, with 100% indicating perfect consistency. Participants who fell outside of a 25% threshold or who were explicitly tapping on the melody rather than the beat were excluded. Calculating the mean percent ratio for each group (PwPD vs. HC) with all of the included participants, we found the means to be 99.51% for PD and 102.92% for controls. These means are similar and very close to 100%, indicating high consistency between mental and music conditions.

Sixty-seven people were screened for the study. Three PwPD did not pass screening. We also had two PwPD and one HC unable to do the MRI due to claustrophobia or tremor. Five additional HC were excluded from analysis for tapping inconsistencies (see above). The final analysis included 27 PwPD (66.8 (±6.02) years) and 28 HC (66.46 (±9.39) years; Table 1).

**Table 1:** Participant demographics.

	Healthy controls (*n* = 28)	PwPD (*n* = 27)
Sex, % male	32.14%	25.93%
Age, years	66.46 (9.39)	66.8 (6.02)
MDS-UPDRS-III, mean (SD)	–	35.26 (13.3)
H&Y	–	All stage II
LEDD, mg^*^	–	602.38
MMSE, median (range)	29 (26,30)	29 (27,30)
Fallers (%)	36%	26%

LEDD, levodopa equivalent daily dose (* = two individuals were not taking this medication); MDS-UPDRS-III: Movement Disorders Society-Unified Parkinson Disease Rating Scale Part III; MMSE: Mini-Mental Status Examination; Fallers: % of participants who reported 1 or more falls in the past year; PwPD, People with Parkinson disease.

#### 2.2.5 Processing

During and after acquisition, images were visually inspected for artifact. T1 images were processed through FreeSurfer version 7.1 for parcellation and segmentation of cortical and subcortical regions, respectively, (Fischl and Dale, 2000; Fischl et al., 2002; Fischl et al., 2004). Following this, the preprocessing pipeline was completed, including distortion correction, slice timing correction, motion correction, temporal filtering, registration, and normalization (Raut et al., 2019). Preprocessed images were then further processed and statistical tests run in Statistical Parametric Mapping (SPM).

##### 2.2.5.1 BOLD preprocessing

Standard preprocessing techniques were used to perform slice-timing correction, motion correction, bias field correction, distortion correction, FNIRT non-linear registration (Andersson et al., 2024), and normalization (Shulman et al., 2010). During this preprocessing, individual participant’s fMRI images were registered to their T1-weighted image, which was aligned to MNI space (Mai and Majtanik, 2017). Functional images were smoothed by a Gaussian filter with a full width half max of 6 mm. Visual QC was performed throughout these steps. Scans with greater than 25% of frames exceeding 0.5 mm frame wise displacement were excluded to maintain low motion data (4 participants, 1 run each) (Hagler et al., 2019).

##### 2.2.5.2 Processing in SPM

Following standard preprocessing steps, brain activity was analyzed using SPM12 (Friston et al., 2007). Beta weight contrasts for each participant’s task effects (i.e., subtraction of rest portions from task blocks seen in Figure 2) were estimated using a general linear model (GLM) taking into account the canonical hemodynamic response (Friston et al., 1995). These individual task effects were then used for between group (PwPD vs. HC) and full cohort (all participants) analyses. Task effects, comparing the task block to the rest block, for Tap, Tap+Music, and Tap+Mental conditions were calculated. Then, condition effects, comparing task effect results between conditions, for mentally singing (Tap+Mental – Tap) and listening to music (Tap+Music – Tap), and differences between Tap+Mental and Tap+Music conditions were analyzed. Each of these analyses were performed across the whole brain and with hypothesis driven putamen, anterior cerebellum, and posterior cerebellum regions of interest (ROI). ROIs were created using WFUPickAtlas toolbox in SPM12 (Maldjian et al., 2003). Family wise error (FWE) correction was used to identify significant areas, or “clusters,” of brain activity.

##### 2.2.5.3 Sample size determination

Previous MRI studies showed significant differences in fMRI brain activity with different types of cues (e.g., visual vs. auditory) with sample sizes of just 10–15 participants (Hove et al., 2013; Pollok et al., 2009; Karabanov et al., 2009). Our own prior work showed differences in brain activity during imagined walking tasks between people with and without freezing of gait with only 10 people per group, and between PD and control groups on imagined gait tasks with 20 people per group (Peterson et al., 2014; Peterson et al., 2014). Our preliminary data from PwPD (*n* = 10) showed large effect sizes for both of our variability outcomes with a mean difference of 0.69 (SE = 0.12) between the music and mental singing conditions for stride time variability. To detect a moderate effect size (Cohen’s d = 0.5) in a RM ANOVA with 2 groups and 3 conditions at 80% power, a total sample size of 50 would be needed. As such, we recruited 25 people per group as sufficient to provide adequate power.

## 3 Results

### 3.1 MRI

Significant results from fMRI analysis in SPM12 are summarized in Tables 2, 3. There were no significant differences identified when investigating task or condition effects between groups in any comparison; thus, groups were combined to increase power through full cohort analysis.

**Table 2:** Significant task effect results by condition.

	Cluster				Peak			MNI coordinates			Anatomical Regions	
	#	*p* (FWE)	size (voxels)	size (mL)	*p* (FWE)	T	equivZ	x	y	z	AAL label	BA Label
Tap task effect (Figure 3A)	1	**<0.001**	**262**	**7.074**	**0.003**	**6.15**	**5.33**	**−38**	**−24**	**48**	**Postcentral_L**	**Left-PrimSensory (1)**
					**0.038**	**5.34**	**4.77**	**−50**	**−18**	**54**	**Postcentral_L**	**Left-PrimSensory (1)**
					0.068	5.15	4.62	−44	−21	60	Postcentral_L	Left-PrimMotor (4)
					0.091	5.05	4.55	−38	−30	66	Postcentral_L	Left-PrimMotor (4)
					0.167	4.83	4.38	−56	−21	45	Parietal_Inf_L	Left-BA40
					0.212	4.74	4.31	−32	−33	51	Postcentral_L	Left-PrimSensory (1)
					0.496	4.36	4.02	−28	−30	69	Postcentral_L	Left-PrimSensory (1)
					0.67	4.18	3.87	−52	−12	39	Postcentral_L	Left-PrimMotor (4)
					0.991	3.6	3.39	−32	−24	69	Precentral_L	Left-PrimMotor (4)
Tap + Music task effect (Figure 3B)	1	**<0.001**	**405**	**10.935**	**0**	**8.66**	**6.81**	**−38**	**−24**	**51**	**Postcentral_L**	**Left-PrimSensory (1)**
					**0**	**7.67**	**6.26**	**−46**	**−21**	**48**	**Postcentral_L**	**Left-PrimSensory (1)**
					**0.001**	**6.47**	**5.53**	**−32**	**−33**	**54**	**Postcentral_L**	**Left-PrimSensory (1)**
					**0.033**	**5.43**	**4.82**	**−32**	**−30**	**66**	**Postcentral_L**	**Left-PrimSensory (1)**
					0.9	3.92	3.66	−20	−33	72	Postcentral_L	Left-PrimSensory (1)
					0.941	3.84	3.59	−16	−36	69	Precuneus_L	Left-PrimSensory (1)
	2	**<0.001**	**637**	**17.199**	**0**	**6.85**	**5.77**	**−50**	**−9**	**0**	**Temporal_Sup_L**	**Left-PrimAuditory (41)**
					**0**	**6.69**	**5.67**	**−52**	**−39**	**12**	**Temporal_Mid_L**	**Left-BA22**
					**0.006**	**5.95**	**5.18**	**−56**	**−24**	**6**	**Temporal_Sup_L**	**Left-PrimAuditory (41)**
					**0.008**	**5.89**	**5.14**	**−38**	**−36**	**12**	**Temporal_Sup_L**	**Left-PrimAuditory (41)**
					0.304	4.62	4.21	−28	−15	3	Putamen_L	Left-Putamen (49)
	3	**<0.001**	**478**	**12.906**	**0**	**6.76**	**5.71**	**50**	**−6**	**0**	**Temporal_Sup_R**	**Right-PrimAuditory (41)**
					**0**	**6.7**	**5.68**	**52**	**−9**	**3**	**Temporal_Sup_R**	**Right-PrimAuditory (41)**
					**0.049**	**5.3**	**4.72**	**62**	**−24**	**6**	**Temporal_Sup_R**	**Right-PrimAuditory (41)**
					0.095	5.07	4.55	64	−27	0	Temporal_Mid_R	Right-BA22
					0.1	5.05	4.54	62	0	0	Temporal_Sup_R	Right-BA22
					0.146	4.91	4.44	62	−33	9	Temporal_Sup_R	Right-BA22
					0.367	4.54	4.15	50	−33	12	Temporal_Sup_R	Right-PrimAuditory (41)
					0.532	4.35	4	68	−36	15	Temporal_Sup_R	Right-BA22
					0.995	3.57	3.37	56	9	−6	Temporal_Pole_Sup_R	Right-BA22
	4	**0.001**	**123**	**3.321**	**0.016**	**5.67**	**4.99**	**−10**	**−15**	**60**	**Supp_Motor_Area_L**	**Left-BA6**
	*5*	***0.004***	***43***	***1.161***	***0.004***	***4.62***	***4.21***	***−28***	***−15***	***3***	***Putamen_L***	***Left-Putamen (49)***
Tap + Mental task effect (Figure 3C)	1	**<0.001**	**964**	**26.028**	**0**	**6.67**	**5.67**	**−46**	**−21**	**51**	**Postcentral_L**	**Left-PrimSensory (1)**
					**0.001**	**6.6**	**5.63**	**−50**	**−15**	**54**	**Postcentral_L**	**Left-PrimSensory (1)**
					**0.005**	**5.98**	**5.21**	**−34**	**−27**	**48**	**Postcentral_L**	**Left-PrimSensory (1)**
					**0.018**	**5.56**	**4.92**	**−8**	**−15**	**60**	**Supp_Motor_Area_L**	**Left-BA6**
					**0.039**	**5.31**	**4.74**	**−32**	**−30**	**66**	**Postcentral_L**	**Left-PrimSensory (1)**
					0.15	4.85	4.4	−14	−30	66	Paracentral_Lobule_L	Left-PrimMotor (4)
					0.267	4.62	4.22	4	−30	66	Paracentral_Lobule_R	Right-PrimMotor (4)
					0.344	4.51	4.14	8	−24	66	Paracentral_Lobule_R	Right-BA6
					0.423	4.42	4.06	−16	−27	63	Paracentral_Lobule_L	Left-PrimMotor (4)
					0.606	4.22	3.91	−4	−33	60	Paracentral_Lobule_L	Left-PrimSensory (1)
					0.724	4.1	3.81	4	−9	66	Supp_Motor_Area_R	Right-BA6
					0.786	4.03	3.75	−26	−36	69	Postcentral_L	Left-SensoryAssoc (5)
					0.946	3.77	3.54	−10	−39	57	Precuneus_L	Left-BA31
					0.972	3.69	3.47	−16	−9	48	Cingulum_Mid_L	Left-BA6
					0.992	3.55	3.35	−16	−9	42	Cingulum_Mid_L	Left-BA24
	2	**0.004**	**114**	**3.078**	**0.011**	**5.73**	**5.04**	**−32**	**−12**	**0**	**Putamen_L**	**Left-Putamen (49)**
	*3*	***0.003***	***52***	***1.404***	***0.009***	***4.79***	***4.35***	***10***	***−51***	***−18***	***Cerebelum_4_5_R***	
	*4*	***0.002***	***68***	***1.836***	***0***	***5.73***	***5.04***	***−32***	***−12***	***0***	***Putamen_L***	***Left-Putamen (49)***

All within cluster peaks with T > 3.5 shown, with peaks maintaining significance after FWE multiple comparisons correction shown in bold. Italicized rows indicate results from ROI confined analyses.

**Table 3:** Significant condition effect results by condition.

	Cluster				Peak			MNI coordinates			Anatomical Regions	
	#	*p* (FWE)	size (voxels)	size (mL)	*p* (FWE)	T	equivZ	x	y	z	AAL label	BA Label
Music condition effect (Figure 4A) [(Tap + Music task effect) – (Tap task effect)]	1	**<0.001**	**175**	**4.725**	**0.02**	**5.63**	**4.96**	**−50**	**−18**	**3**	**Temporal_Sup_L**	**Left-PrimAuditory (41)**
					0.054	5.35	4.76	−62	−18	3	Temporal_Sup_L	Left-BA22
					0.106	5.11	4.59	−58	−21	6	Temporal_Sup_L	Left-PrimAuditory (41)
					0.645	4.33	3.99	−46	−3	−9	Temporal_Sup_L	Left-BA22
					0.879	4.06	3.77	−44	18	9	Frontal_Inf_Tri_L	Left-BA44
					0.914	4	3.72	−52	0	−3	Temporal_Sup_L	Left-BA22
					0.958	3.89	3.64	−50	9	0	Temporal_Pole_Sup_L	Left-BA44
					0.997	3.64	3.43	−34	24	9	Insula_L	Left-BA45
	2	<0.001	275	7.425	0.096	5.15	4.61	50	−3	−3	Temporal_Sup_R	Right-BA22
					0.108	5.1	4.58	52	−12	3	Temporal_Sup_R	Right-PrimAuditory (41)
					0.16	4.96	4.48	58	−24	6	Temporal_Sup_R	Right-PrimAuditory (41)
					0.201	4.88	4.41	64	−27	0	Temporal_Mid_R	Right-BA22
					0.324	4.68	4.26	58	−36	9	Temporal_Sup_R	Right-BA22
					0.806	4.16	3.85	52	12	−6	Temporal_Pole_Sup_R	Right-BA38
					0.88	4.06	3.77	50	18	−6	Frontal_Inf_Orb_R	Right-BA47
					0.892	4.04	3.75	50	6	3	Rolandic_Oper_R	Right-BA44
					0.94	3.94	3.68	68	−39	15	Temporal_Sup_R	Right-BA22
					0.968	3.86	3.61	64	−45	6	Temporal_Mid_R	Right-BA21
					0.998	3.62	3.41	56	−45	12	Temporal_Mid_R	Right-BA39
Mental singing condition effect (Figure 4B) [(Tap + Mental task effect) – (Tap task effect)]	1	0.032	59	1.593	0.357	4.58	4.19	−32	−15	3	Putamen_L	Left-Putamen (49)
					0.618	4.29	3.96	−28	−15	9	Putamen_L	Left-Putamen (49)
					0.717	4.19	3.88	−32	−6	12	Insula_L	Left-Insula (13)
	2	0.028	61	1.647	0.658	4.25	3.93	44	−72	0	Temporal_Mid_R	Right-BA19
					0.684	4.22	3.91	44	−78	9	Occipital_Mid_R	Right-BA19
					0.944	3.87	3.62	34	−69	9	Occipital_Mid_R	Right-BA19
	3	0.01	78	2.106	0.948	3.86	3.61	−44	−24	45	Parietal_Inf_L	Left-PrimSensory (1)
					0.976	3.76	3.53	−28	−24	51	Postcentral_L	Left-PrimMotor (4)
					0.992	3.65	3.43	−44	−12	60	Precentral_L	Left-BA6
					0.997	3.57	3.36	−50	−12	45	Postcentral_L	Left-PrimMotor (4)
	*4*	***0.003***	***42***	***1.134***	***0.006***	***4.58***	***4.19***	***−32***	***−15***	***3***	***Putamen_L***	***Left-Putamen (49)***
					***0.013***	***4.29***	***3.96***	***−28***	***−15***	***9***	***Putamen_L***	***Left-Putamen (49)***
Music condition vs mental singing condition (Figure 4C) [(Tap + Music task effect) – (Tap + Mental task effect)]	1	**<0.001**	**1,035**	**27.945**	**0**	**15.6**	**Inf**	**−46**	**−18**	**6**	**Temporal_Sup_L**	**Left-PrimAuditory (41)**
					**0**	**11.8**	**Inf**	**−50**	**−9**	**−3**	**Temporal_Sup_L**	**Left-BA22**
					**0**	**11.3**	**Inf**	**−38**	**−36**	**12**	**Temporal_Sup_L**	**Left-PrimAuditory (41)**
					**0**	**10.9**	**Inf**	**−62**	**−18**	**6**	**Temporal_Sup_L**	**Left-PrimAuditory (41)**
					**0**	**9.86**	**7.4**	**−56**	**−36**	**9**	**Temporal_Mid_L**	**Left-BA22**
					**0**	**9.42**	**7.19**	**−46**	**−3**	**−9**	**Temporal_Sup_L**	**Left-BA22**
					**0**	**9.23**	**7.1**	**−56**	**−33**	**3**	**Temporal_Mid_L**	**Left-BA21**
					0.878	4.05	3.76	−62	−51	27	SupraMarginal_L	Left-BA39
					0.888	4.03	3.75	−62	−48	21	Temporal_Sup_L	Left-BA39
	2	**<0.001**	**1,278**	**34.506**	**0**	**14.5**	**Inf**	**50**	**−12**	**0**	**Temporal_Sup_R**	**Right-PrimAuditory (41)**

All within cluster peaks with T > 3.5 shown, with peaks maintaining significance after FWE multiple comparisons correction shown in bold. Italicized rows indicate results from ROI confined analyses.

#### 3.1.1 Task effects

##### 3.1.1.1 Tap task effect

**Figure 3:**
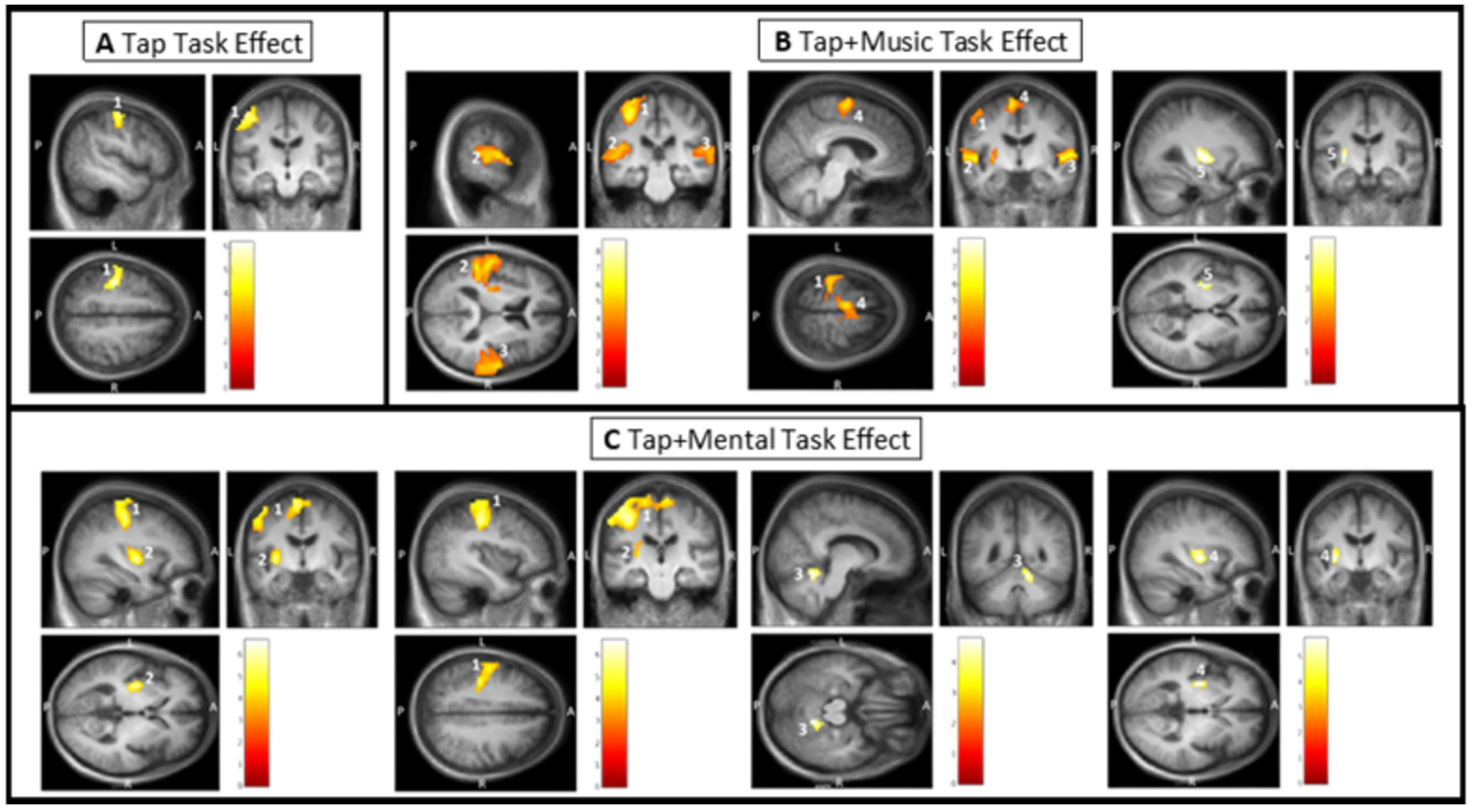
Task effect results from SPM analysis thresholded for significance. Color bars in each window display T values with the lowest being dark red and the highest being white. Clusters are labeled based on their number in Table 1. **(A)** Tap task effect. Cluster 1 covering the left primary sensory cortex and motor cortex. **(B)** Tap + Music task effect. Cluster 1 covering the left primary auditory sensory cortex; Cluster 2 covering the left primary auditory cortex, left superior temporal gyrus, and left putamen; Cluster 3 covering the right primary auditory cortex and right superior temporal gyrus; Cluster 4 covering the left supplementary motor cortex; and Cluster 5 within the left putamen (ROI confined analysis). **(C)** Tap + Mental task effect. Cluster 1 covering the left primary sensory cortex, left supplementary motor area, left primary motor cortex, right supplementary motor cortex, left cingulate cortex, and left postcentral gyrus; Cluster 2 covering a portion of the left putamen; Cluster 3 within the right anterior cerebellum (ROI confined analysis); and Cluster 4 peak within the left putamen (ROI confined analysis).

During the uncued tapping task (Tap), there was one significant cluster of greater activation when compared to rest. This cluster covers the left primary sensory and left motor cortex (Figure 3A; Table 2). Within this cluster, significant peaks of activation were located in the left primary sensory cortex. There were no significant tap task effects in ROI confined analyses.

##### 3.1.1.2 Tap + Music task effect (external cue)

While listening and tapping to music (Tap+Music), there were five significant clusters displaying greater activity than while at rest (Figure 3B; Table 2). The first cluster covers the left primary sensory cortex, with peaks of significant activation located within the left primary sensory cortex. The second cluster covers the left primary auditory cortex, left superior temporal gyrus, and left putamen. This cluster includes significant peaks of activation in the left primary auditory cortex and left superior temporal gyrus. The third cluster covers the right primary auditory cortex and right superior temporal gyrus, with significant peaks within the right auditory cortex, and the fourth cluster includes the left supplementary motor cortex, containing a significant peak of activation within this area of cortex (Figure 3B; Table 2). ROI confined analyses revealed the fifth cluster in the left putamen exhibiting greater activity during the Tap+Music task compared to rest (Figure 3B).

##### 3.1.1.3 Tap + Mental task effect (internal cue)

Tapping while mentally singing (Tap+Mental) displayed greater activity when compared to rest in four significant clusters (Figure 3C; Table 2). The first cluster covers the left primary sensory cortex, left supplementary motor area, left primary motor cortex, right supplementary motor cortex, left cingulate cortex, and left postcentral gyrus. Within this cluster, significant peaks of activation are located in the left primary sensory cortex and left supplementary motor area. The second cluster is in the left putamen. ROI confined analyses revealed the third cluster in the right anterior cerebellum and the fourth cluster in the left putamen also exhibiting greater activity in the Tap+Mental task compared to rest (Figure 3C; Table 2).

#### 3.1.2 Condition effects

##### 3.1.2.1 Music condition effect [(Tap + Music task effect) – (Tap task effect)] (external cue)

**Figure 4:**
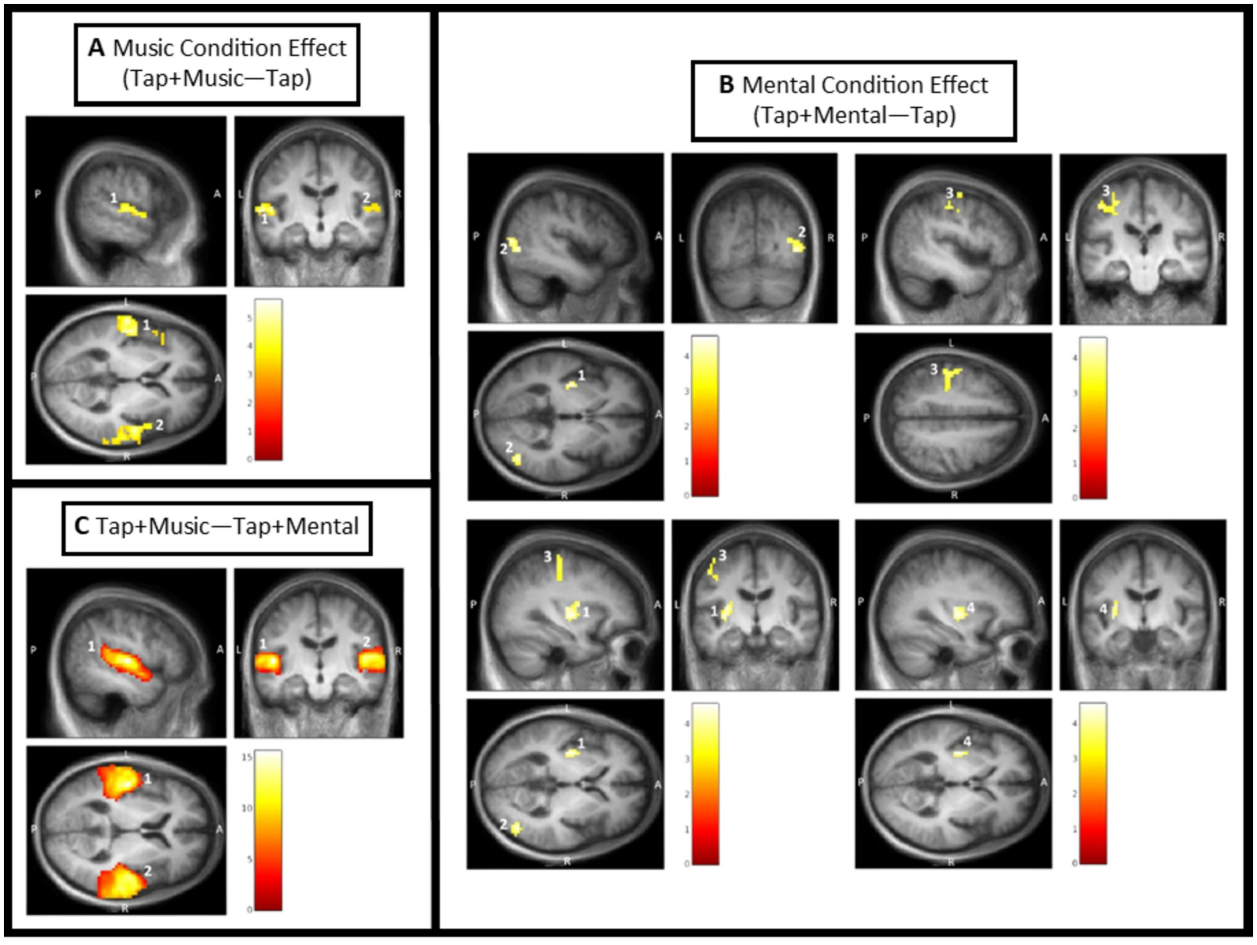
Condition effect results from SPM analysis thresholded for significance. Color bars in each window display T values with the lowest being dark red, and the highest being white. Clusters are labeled based on their number in Table 2. **(A)** Music condition effect [(Tap + Music) – Tap]. Cluster 1 covering the left primary auditory cortex, left superior temporal gyrus, and left insula; and Cluster 2 covering the right superior temporal gyrus, right primary auditory cortex, right temporal pole, right frontal cortex, and right angular gyrus. **(B)** Mental condition effect [(Tap + Mental) – Tap]. Cluster 1 covering the left putamen and left insula; Cluster 2 covering the right associative visual cortex; Cluster 3 covering the left primary sensory cortex, left primary motor cortex, and left premotor cortex; and Cluster 4 within the left putamen (ROI confined analysis). **(C)** Difference between Tap + Music and Tap + Mental conditions [(Tap + Music) – (Tap + Mental)]. Cluster 1 covering the left primary auditory cortex, left superior temporal gyrus, left middle temporal gyrus, and left angular gyrus; and Cluster 2 covering the right primary auditory cortex.

The music condition effect exhibited two significant clusters of activation (Figure 4A; Table 3). The first cluster is composed of the left primary auditory cortex, left superior temporal gyrus, and left insula. A significant peak of activation in this cluster was located in the left primary auditory cortex. The second cluster covers the right superior temporal gyrus, right primary auditory cortex, right temporal pole, right frontal cortex, and right angular gyrus, with no significant individual peaks of activation within this cluster (Figure 4A; Table 3). There were no significant music condition effects in ROI confined analyses.

##### 3.1.2.2 Mental singing condition effect [(Tap + Mental task effect) – (Tap task effect)] (internal cue)

There were four significant clusters of greater activation during the Tap+Mental task compared to the Tap only task (Figure 4B; Table 3). The first cluster includes the left putamen and left insula and the second cluster is primarily composed of the right associative visual cortex. The third cluster covers the left primary sensory cortex, left primary motor cortex, and left premotor cortex. There were no significant individual peaks in any of these three clusters. ROI confined analyses revealed the fourth significant cluster in the left putamen also exhibiting activation from the mental signing condition effect.

##### 3.1.2.3 Music condition compared to Mental singing condition [(Tap + Music task effect) (Tap + Mental task effect)]

The Tap+Music condition displayed greater activity than the Tap+Mental condition in two significant clusters (Figure 4C; Table 3). The first cluster covers the left primary auditory cortex, left superior temporal gyrus, left middle temporal gyrus, and left angular gyrus. Within this cluster, significant peaks of activation were in the left primary auditory cortex, left superior temporal gyrus, and left middle temporal gyrus. The second cluster includes the right primary auditory cortex, with a significant peak of activation within this cortex. There were no significant areas of greater activation in Tap+Music than Tap+Mental identified in ROI confined analyses. Furthermore, there were no significant areas of increased activity during the Tap+Mental condition compared to the Tap+Music condition in full brain or ROI analyses.

## 4 Discussion

The goal of this study was to investigate the brain mechanisms underlying acute responses to external and internal musical cues in PwPD and older adults. Our hypotheses were only partially substantiated. Brain activity during external and internal cueing confirmed utilization of some parts of each of the hypothesized pathways, but the distinctions were not as clear or straightforward as we expected. We also saw no significant differences between groups, suggesting similarities in brain activation patterns during cues for both PwPD and older adults.

### 4.1 Differences between cue types

Matching movement to external cues requires perceiving auditory information, temporally predicting future beats, and synchronizing movement to an outside source. Confirming the task was working, we saw significant bilateral auditory cortex activity when external musical cues were playing. We expected to see more activity in the CTC pathway during external cues. Internal cues require perceiving an auditory stimulus, which primes the motor system, and then continuing the beat in your own head once the stimulus is removed. Self-paced tapping is thought to require more neural resources and is associated with prefrontal cortex activation suggesting increased attentional demands (De Pretto et al., 2018). We expected to see more activity in the SPT pathway during internal cues.

In the task effects, we saw several commonalities between cue types. Whole brain analyses revealed activations of primary motor and primary sensory cortices. Both external and internal cues activated motor (SMA) and sensory (postcentral gyrus) areas, which confirm strong connections between sensorimotor areas during cued rhythmic movements (Cerasa et al., 2006; Mak et al., 2016). Activations of the left postcentral gyrus may reflect its role in processing external rhythmic tempos (Thibault et al., 2023), but it is also partly activated by imagining songs (Gunji et al., 2007). The SMA has dense connections with M1 and is associated with sensorimotor and sequential temporal processing (Braunlich et al., 2019). The SMA is implicated in motor planning during beat perception and production, particularly for complex rhythms (Patel et al., 2005). SMA activity is common in beat continuation conditions, which highlights its role in explicit timing of repetitive movements (Elsinger et al., 2003).

We also saw significant basal ganglia activity during cueing. Whole-brain and ROI-based analyses of task effects showed putamen activation during both cue types, which suggests striatal involvement while matching movement to both external and internal beats. In the condition effects, putamen activity was only noted during internal cueing. Anatomically, the striatum receives direct projections from auditory cortical regions, which could support auditory entrainment and the effects of rhythmic auditory stimulation. The putamen is particularly implicated in beat-based rhythmic processing during internal cues (Taniwaki et al., 2013), but a meta-analysis of studies on healthy adults found the putamen was equally active in both external and internal pacing conditions whereas the globus pallidus, an output nuclei, was more active during internal cueing (Chauvigné et al., 2014). Overall, activity within the SMA-basal ganglia loop during both cue types suggests both may at least partially work through the striato-pallado-thalamocortical (SPT) pathway.

We saw cerebellar activations during internal but not external cueing. The cerebellum receives direct projections for auditory cortical regions and plays a role in automatic encoding with high temporal precision (Martinu and Monchi, 2013). In healthy adults, previous research has noted a more active cerebellum during externally-cued tasks than at rest, as external cues elicit stronger cerebellar connectivity to the premotor cortex via the thalamus (Taniwaki et al., 2013; Chauvigné et al., 2014). As such, we expected that external cues would elicit greater activation of the cerebellar-thalamo-cortical (CTC) pathway, but instead significant cerebellar activation only occurred during *internal* cueing. Though this contrasts with literature recognizing a specific cerebellar role in externally-cued tasks, we are not the first to report cerebellar recruitment for internally-cued tasks. Past fMRI studies of internally-cued movement show regional activations of the anterior and inferior cerebellum (Schlerf et al., 2010). Another study of self-priming cues showed overactivation of cerebellar loops in PwPD specifically (Li et al., 2021). A recent study showed cerebellar recruitment for both PwPD and HC in external cuing, but only for PwPD in internal cueing (Drucker et al., 2019). In their study, they also saw increased recruitment of ipsilateral (right) cerebellum for PwPD. Cerebellar activation during internal cueing may warrant further research.

Our external cue was performed on a piano, while the internal cue consisted of imagined singing with lyrics. Thus, the internal cues contained lyrics whereas the external cue did not. Brain activation may differ with different types of auditory stimuli, and singing may present an intermediate condition between instrumental music and speech. Past work shows music preferentially engages the superior temporal gyrus, while the human voice, either spoken or sung, activates it even more strongly (Whitehead and Armony, 2018). This region contains Wernicke’s area, Brodmann area 22, an important region for understanding speech. Our results contrast this as they show activation of the left superior temporal gyrus only in the music condition. In the music condition, we also saw activation of the pars opercularis (BA 44) and the pars triangularis (BA 45), both areas that play a critical role in language production.

Two other differences should be noted. The Music condition effect exhibited a cluster of activation that included the frontal cortex. Previously, similar increases in prefrontal cortex activity have been reported when walking with external cues (Vitorio et al., 2018). This activation could reflect the executive/attentional control needed to synchronize footsteps to an external cue. The Mental condition exhibited right associative visual cortex activity, which could be related to the need to watch the screen for directions because there was no sound cue for any trial but the first.

### 4.2 Differences between groups

Past fMRI studies suggest PwPD inadequately activate dopamine-dependent STC circuitry during internally-generated tasks leading to compensatory recruitment of spared CTC circuits (Sen et al., 2010). This compensatory pathway is thought to account for the suitability of rhythmic auditory stimulation for PwPD, as people are able to use RAS as a template to restore the lost sense of rhythmicity that normally relies on the proper striatal functioning (Braunlich et al., 2019). Internal cues, in contrast, are thought to rely more on the SPT pathway, which would suggest they were less available to PwPD and therefore less effective as a therapy to improve gait.

Our results do not fit into this framework. We saw no differences between groups in any of the task or condition effects. Several reasons could account for this.

First, our participant sample could have been too mild in disease severity to experience significant dopamine depletion within the basal ganglia. If this area is less affected, then it is possible there was no need to reroute around it at this stage of the disease. Our participants were also on their normal dopaminergic medication, which may have restored corticostriatal activity levels enough to reduce the impact of cues on brain activity (Drucker et al., 2019).

Second, an alternative theory to the one we proposed is that both external and internal cues work via facilitation of remaining function within the SPT loop. As we saw activation of the putamen and SMA during both cue types, this theory presents a viable explanation for how cues may boost any preserved function in degenerated areas.

Third, our results could relate to the priming method utilized in our paradigm. Since our priming method was designed to create consistency between tasks, it may have inadvertently contributed to similarities seen between tasks. Many internal cueing paradigms do not offer a priming cue before starting the task; however, in our task, people first heard the music and then continued the task in silence. Similar priming effects—in beat continuation studies, for example—have shown reliance on cerebellar loops to compensate for basal ganglia dysfunction (Li et al., 2021). When measuring brain activity during movement timing tasks, a challenge exists in teasing apart when cued timing transitions to emergent timing within the participant. The cerebellum plays a role in coordinating absolute timing to perceived timing via sensory feedback loops, and that role may be even more important during internal cues as a participant is attempting to synchronize internal timing to their own emergent perceptions of absolute timing (Grahn and Rowe, 2013).

An important consideration when interpreting our results is that CTC and SPT circuits are anatomically and functionally related. PD progression influences both, although cerebellar degeneration is less commonly reported than striatal degeneration. In a study that followed PwPD and HC over a two-year period, both groups exhibited degeneration in CTC circuits (Sen et al., 2010). Only PwPD, however, increased recruitment of cortical motor and cerebellar regions during internally-generated finger movements. This suggests internal cues may facilitate activity in multiple impaired regions and could account for the neural activations we saw in both circuits during mental singing.

### 4.3 Therapeutic application of external and internal cues

In our gait laboratory, we have shown both external and internal cues have immediate effects on gait in PwPD and HC (Harrison et al., 2017; Harrison et al., 2018; Harrison et al., 2019; Horin et al., 2020). Gait benefits from rhythmic cues suggest that, in spite of basal ganglia degeneration, PwPD are largely able to utilize cueing techniques. Brain activity patterns seen in this study tell a similar story: that both cue types are mechanistically similar, routing through parallel pathways and utilizing portions of both the SPT and CTC pathways.

In laboratory settings, we found internal cues reduce gait variability more than external cues (Harrison et al., 2019). We previously theorized that higher variability during external cueing relates to the challenge of matching movement to an external source. The neural patterns during external cueing show that auditory information that allows for auditory-motor coupling is accompanied by other sensory inputs as well. Potentially, during external cueing, many inputs are being processed simultaneously which challenges the nervous system to synchronize motor outputs. During internal cueing, on the other hand, less auditory information coming in could potentially free up resources to focus on task initiation and precision. Internal cues activated cerebellar pathways, which may facilitate motor preparation and self-timing. Cerebellar activity during mental singing may help explain why this cue type typically elicits reductions in movement variability (i.e., more even movement during self-paced motor synchronization).

Much of what is currently known regarding the mechanisms of internal cues is based on research into self-generated attentional cues or rhythmic continuation tasks, in which an auditory, visual, or tactile cue is removed. Little is known about the patterns of brain activation that occur during mental singing, specifically. Only one study that we know of explored brain activations during mental singing and found similar activity as during singing aloud (Kleber et al., 2007). Singing aloud results in activation of several areas within the frontal, parietal, and temporal cortices along with subcortical and brainstem structures including pre-SMA, SMA, dorsolateral prefrontal cortex, primary somatosensory and auditory cortices, basal ganglia, and cerebellum (Mavridis and Pyrgelis, 2016). In our study of mental singing, we also saw widespread activation of these areas, which further promotes the idea that mental singing can be used covertly to cue movement as readily as overt singing aloud.

We do not know for sure if mental singing functions like other internal cueing tasks. Many internally-cued movements are done in isolation with one effector. For instance, a unimanual finger tap, commonly used in Finger Tapping Tasks (FTT), might be cued once and then continued rhythmically with attentional resources only. Mental singing may require integration of many other tasks aside from maintaining rhythm. When mental singing, one is asked to recall all the musical elements of a song, including rhythm, melody, lyrics, dynamics, and phrasing. One might even feel like they are hearing their own voice in their head and respond to feedback from the imagined perception of it (Zarate, 2013). Attentional demands may be higher in internal cueing rather than external cueing because the onus to complete the mental task falls within the person performing the internal cue. However, mental singing may also reduce attentional load because of the ease of matching movement to a song that may be stored in long-term memory and thus readily retrievable.

### 4.4 Limitations

These results should be interpreted in light of some limitations. Our sample was mild in disease severity and tested on medication, so we do not know how these results would translate to those with higher levels of impairment or to the off-medication state. People with PD with higher disease severity are known to exhibit higher recruitment of CTC networks during cued movements, so testing a more impaired population may have altered our results (Sen et al., 2010).

We also chose to test participants on medication to avoid methodological difficulties of increased tremor movement inside the scanner. Dopaminergic medication can increase overall BOLD activity and thereby reduce ability to detect the effects of RAS on the SPT network (Elsinger et al., 2003). Though we do not know how these results would translate to the off-medication state, even greater motor benefits have been seen during RAS when there is more room for improvement (Erra et al., 2019).

We only imaged participants while finger tapping, and although we have previously shown this is a strong proxy for gait (Horin et al., 2021), we cannot know how accurately these conclusions translate to brain activity during actual walking tasks. Differences in premotor connectivity during unimanual and foot tapping tasks have been reported, with hand movements revealing stronger cortical representations in motor planning regions (Drucker et al., 2019). It is not clear from our results if we would see the same activations during lower extremity movement.

Another factor to consider when interpreting our results is that our tapping rates were determined based on the uncued tapping tempo which may not have aligned perfectly with uncued gait cadence. Participants’ personalized cue rate was then adjusted based on the percentage of gait optimization. This design resulted in a range of bpm of optimized cue rates (mean bpm = 118.5 (±18.96)).

Lastly, human movement is rarely isolated into external and internal cues. Rather, externally cued and internally cued movement often work in tandem or fluidly transfer back and forth. Understanding the neural mechanisms behind each cue type can help determine the most effective approach to integrating these techniques into future rehabilitative interventions.

## 5 Conclusion

This study is the first to elucidate the neural mechanisms underlying internal musical cuing in the form of mental singing. In our comparison of mental singing to external musical cueing, we found many similarities in brain activity between cue types. We found no between group differences, suggesting both cue types function similarly for both PwPD and healthy adults. Our results suggest cues activate parts of both the CTC and SPT pathways which may facilitate sensorimotor activity via complex interplay between neural circuits. Internal cues do not seem to require vastly different neural resources than external cues, which implies they are accessible even for people with neurological impairment. These findings have implications for how external and internal cues may be administered in future therapies for PwPD or other populations.

## Data Availability

The raw data supporting the conclusions of this article will be made available by the authors, without undue reservation.

## References

[ref1] Andersson J. O. Jenkinson M. Smith S. Andersson J. L. Andersson J. L. Jenkinson M. *Non-linear registration aka spatial normalisation FMRIB Technial report TR07JA2. – Science open*. (2024). Available online at: https://www.scienceopen.com/book?vid=13f3b9a9-6e99-4ae7-bea2-c1bf0af8ca6e (Accessed December 13, 2024).

[ref2] Bengtsson S. L. Ullén F. Ehrsson H. H. Hashimoto T. Kito T. Naito E.. (2009). Listening to rhythms activates motor and premotor cortices. Cortex 45, 62–71. doi: 10.1016/j.cortex.2008.07.002, PMID: 19041965

[ref3] Bijsterbosch J. D. Lee K. H. Hunter M. D. Tsoi D. T. Lankappa S. Wilkinson I. D.. (2011). The role of the cerebellum in sub-and supraliminal error correction during sensorimotor synchronization: evidence from fMRI and TMS. J. Cogn. Neurosci. 23, 1100–1112. doi: 10.1162/jocn.2010.21506, PMID: 20465354

[ref4] Braunlich K. Seger C. A. Jentink K. G. Buard I. Kluger B. M. Thaut M. H. (2019). Rhythmic auditory cues shape neural network recruitment in Parkinson’s disease during repetitive motor behavior. Eur. J. Neurosci. 49, 849–858. doi: 10.1111/ejn.14227, PMID: 30375083 PMC6426668

[ref5] Cerasa A. Hagberg G. E. Peppe A. Bianciardi M. Gioia M. C. Costa A.. (2006). Functional changes in the activity of cerebellum and frontostriatal regions during externally and internally timed movement in Parkinson’s disease. Brain Res. Bull. 71, 259–269. doi: 10.1016/j.brainresbull.2006.09.014, PMID: 17113955

[ref6] Chauvigné L. A. S. Gitau K. M. Brown S. (2014). The neural basis of audiomotor entrainment: an ALE meta-analysis. Front. Hum. Neurosci. 8:776. doi: 10.3389/fnhum.2014.00776, PMID: 25324765 PMC4179708

[ref7] Chen J. L. Penhune V. B. Zatorre R. J. (2008). Listening to musical rhythms recruits motor regions of the brain. Cereb Cortex 18, 2844–2854. doi: 10.1093/cercor/bhn042 18388350

[ref8] Chen J. L. Zatorre R. J. Penhune V. B. (2006). Interactions between auditory and dorsal premotor cortex during synchronization to musical rhythms. Neuroimage 32, 1771–1781. doi: 10.1016/j.neuroimage.2006.04.207, PMID: 16777432

[ref9] de Bruin N. Doan J. B. Turnbull G. Suchowersky O. Bonfield S. Hu B.. (2010). Walking with music is a safe and viable tool for gait training in Parkinson’s disease: the effect of a 13-week feasibility study on single and dual task walking. Parkinsons Dis. 2010:483530. doi: 10.4061/2010/483530, PMID: 20976086 PMC2957229

[ref10] De Pretto M. Deiber M. P. James C. E. (2018). Steady-state evoked potentials distinguish brain mechanisms of self-paced versus synchronization finger tapping. Hum. Mov. Sci. 61, 151–166. doi: 10.1016/j.humov.2018.07.007, PMID: 30098488

[ref11] Disbrow E. A. Sigvardt K. A. Franz E. A. Turner R. S. Russo K. A. Hinkley L. B.. (2013). Movement activation and inhibition in Parkinson’s disease: a functional imaging study. J. Parkinsons Dis. 3, 181–192. doi: 10.3233/JPD-130181, PMID: 23938347 PMC4586119

[ref12] Drucker J. H. Sathian K. Crosson B. Krishnamurthy V. McGregor K. Bozzorg A.. (2019). Internally guided lower limb movement recruits compensatory cerebellar activity in people with Parkinson’s disease. Front. Neurol. 10:10. doi: 10.3389/fneur.2019.00537, PMID: 31231297 PMC6566131

[ref13] Elsinger C. L. Rao S. M. Zimbelman J. L. Reynolds N. C. Blindauer K. A. Hoffmann R. G. (2003). Neural basis for impaired time reproduction in Parkinson’s disease: an fMRI study. J Int Neuropsychol Soc 9, 1088–1098. doi: 10.1017/S1355617703970123, PMID: 14738289

[ref14] Erra C. Mileti I. Germanotta M. Petracca M. Imbimbo I. de Biase A.. (2019). Immediate effects of rhythmic auditory stimulation on gait kinematics in Parkinson’s disease ON/OFF medication. Clin. Neurophysiol. 130, 1789–1797. doi: 10.1016/j.clinph.2019.07.013, PMID: 31401487

[ref15] Fischl B. Dale A. M. (2000). Measuring the thickness of the human cerebral cortex from magnetic resonance images. Proc. Natl. Acad. Sci. USA 97, 11050–11055. doi: 10.1073/pnas.200033797, PMID: 10984517 PMC27146

[ref16] Fischl B. Salat D. H. Busa E. Albert M. Dieterich M. Haselgrove C.. (2002). Whole brain segmentation: automated labeling of neuroanatomical structures in the human brain. Neuron 33, 341–355. doi: 10.1016/s0896-6273(02)00569-x 11832223

[ref17] Fischl B. Salat D. H. van der Kouwe A. J. W. Makris N. Ségonne F. Quinn B. T.. (2004). Sequence-independent segmentation of magnetic resonance images. Neuroimage 23, S69–S84. doi: 10.1016/j.neuroimage.2004.07.016, PMID: 15501102

[ref18] Friston K. J. Ashburner J. Kiebel S. Nichols T. Penny W. D. (2007). Statistical parametric mapping: The analysis of Funtional brain images. 1st Edn. Amsterdam, Netherlands: Elsevier/Academic Press.

[ref19] Friston K. J. Frith C. D. Turner R. Frackowiak R. S. (1995). Characterizing evoked hemodynamics with fMRI. Neuroimage 2, 157–165. doi: 10.1006/nimg.1995.1018, PMID: 9343598

[ref20] Ghai S. Ghai I. Schmitz G. Effenberg A. O. (2018). Effect of rhythmic auditory cueing on parkinsonian gait: a systematic review and meta-analysis. Sci. Rep. 8:506. doi: 10.1038/s41598-017-16232-5, PMID: 29323122 PMC5764963

[ref21] Goetz C. G. Tilley B. C. Shaftman S. R. Stebbins G. T. Fahn S. Martinez-Martin P.. (2008). Movement Disorder Society-sponsored revision of the unified Parkinson’s disease rating scale (MDS-UPDRS): scale presentation and clinimetric testing results. Mov. Disord. 23, 2129–2170. doi: 10.1002/mds.22340, PMID: 19025984

[ref22] Grahn J. A. Brett M. (2007). Rhythm and beat perception in motor areas of the brain. J. Cogn. Neurosci. 19, 893–906. doi: 10.1162/jocn.2007.19.5.893, PMID: 17488212

[ref23] Grahn J. A. Rowe J. B. (2009). Feeling the beat: premotor and striatal interactions in musicians and nonmusicians during beat perception. J. Neurosci. 29, 7540–7548. doi: 10.1523/JNEUROSCI.2018-08.2009, PMID: 19515922 PMC2702750

[ref24] Grahn J. A. Rowe J. B. (2013). Finding and feeling the musical beat: striatal dissociations between detection and prediction of regularity. Cereb. Cortex 23, 913–921. doi: 10.1093/cercor/bhs083, PMID: 22499797 PMC3593578

[ref25] Gunji A. Ishii R. Chau W. Kakigi R. Pantev C. (2007). Rhythmic brain activities related to singing in humans. Neuroimage 34, 426–434. doi: 10.1016/j.neuroimage.2006.07.018, PMID: 17049276

[ref26] Hagler D. J. Hatton S. Cornejo M. D.. (2019). Image processing and analysis methods for the adolescent brain cognitive development study. Neuroimage 202:116091. doi: 10.1016/j.neuroimage.2019.116091, PMID: 31415884 PMC6981278

[ref27] Harrison E. C. Tueth L. E. Haussler A. M. Rawson K. S. Earhart G. M. (2025). Personalized auditory rhythmic cues to optimize gait in older adults and people with Parkinson disease. J. Neurol. Phys. Ther. doi: 10.1097/NPT.0000000000000508, PMID: 39913311 PMC12188473

[ref28] Harrison E. C. Horin A. P. Earhart G. M. (2018). Internal cueing improves gait more than external cueing in healthy adults and people with Parkinson disease. Sci. Rep. 8:15525. doi: 10.1038/s41598-018-33942-6, PMID: 30341367 PMC6195608

[ref29] Harrison E. C. Horin A. P. Earhart G. M. (2019). Mental singing reduces gait variability more than music listening for healthy older adults and people with Parkinson disease. J. Neurol. Phys. Ther. 43, 204–211. doi: 10.1097/NPT.0000000000000288, PMID: 31449178 PMC6744333

[ref30] Harrison E. C. McNeely M. E. Earhart G. M. (2017). The feasibility of singing to improve gait in Parkinson disease. Gait Posture 53, 224–229. doi: 10.1016/j.gaitpost.2017.02.008, PMID: 28226309 PMC5373799

[ref31] Hoehn M. M. Yahr M. D. (1967). Parkinsonism: onset, progression, and mortality. Neurology 17, 427–442. doi: 10.1212/wnl.17.5.427 6067254

[ref32] Horin A. P. Harrison E. C. Rawson K. S. Earhart G. M. (2020). People with Parkinson disease with and without freezing of gait respond similarly to external and self-generated cues. Gait Posture 82, 161–166. doi: 10.1016/j.gaitpost.2020.09.005, PMID: 32932076 PMC7718283

[ref33] Horin A. P. Harrison E. C. Rawson K. S. Earhart G. M. (2021). Finger tapping as a proxy for gait: similar effects on movement variability during external and self-generated cueing in people with Parkinson’s disease and healthy older adults. Ann. Phys. Rehabil. Med. 64:101402. doi: 10.1016/j.rehab.2020.05.009, PMID: 32535169

[ref34] Hove M. J. Fairhurst M. T. Kotz S. A. Keller P. E. (2013). Synchronizing with auditory and visual rhythms: an fMRI assessment of modality differences and modality appropriateness. Neuroimage 67, 313–321. doi: 10.1016/j.neuroimage.2012.11.032, PMID: 23207574

[ref35] Karabanov A. Blom O. Forsman L. Ullén F. (2009). The dorsal auditory pathway is involved in performance of both visual and auditory rhythms. Neuroimage 44, 480–488. doi: 10.1016/j.neuroimage.2008.08.047, PMID: 18848999

[ref36] Kleber B. Birbaumer N. Veit R. Trevorrow T. Lotze M. (2007). Overt and imagined singing of an Italian aria. Neuroimage 36, 889–900. doi: 10.1016/j.neuroimage.2007.02.053, PMID: 17478107

[ref37] Leman M. Moelants D. Varewyck M. Styns F. van Noorden L. Martens J. P. (2013). Activating and relaxing music entrains the speed of beat synchronized walking. PLoS One 8:e67932. doi: 10.1371/journal.pone.0067932, PMID: 23874469 PMC3707869

[ref38] Li J. Liu Z. Du Z. Zhu N. Qiu X. Xu X. (2021). Cortical activation during finger tapping task performance in Parkinson’s disease is influenced by priming conditions: an ALE Meta-analysis. Front. Hum. Neurosci. 15:15. doi: 10.3389/fnhum.2021.774656, PMID: 34916919 PMC8669914

[ref39] Mai J. K. Majtanik M. (2017). Human brain in standard MNI space: A comprehensive pocket atlas. Amsterdam, Netherlands: Elsevier.

[ref40] Mak M. K. Y. Cheung V. Ma S. Lu Z. L. Wang D. Lou W.. (2016). Increased cognitive control during execution of finger tap movement in people with Parkinson’s disease. J. Parkinsons Dis. 6, 639–650. doi: 10.3233/JPD-160849, PMID: 27372216

[ref41] Maldjian J. A. Laurienti P. J. Kraft R. A. Burdette J. H. (2003). An automated method for neuroanatomic and cytoarchitectonic atlas-based interrogation of fMRI data sets. Neuroimage 19, 1233–1239. doi: 10.1016/s1053-8119(03)00169-1, PMID: 12880848

[ref42] Martinu K. Monchi O. (2013). Cortico-basal ganglia and cortico-cerebellar circuits in Parkinson’s disease: pathophysiology or compensation? Behav. Neurosci. 127, 222–236. doi: 10.1037/a0031226, PMID: 23244290

[ref43] Mavridis I. N. Pyrgelis E. S. (2016). Brain activation during singing: “clef de sol activation” is the “concert” of the human brain. Med. Probl. Perform. Art. 31, 45–50. doi: 10.21091/mppa.2016.1008, PMID: 26966964

[ref44] McIntosh G. C. (1997). Rhythmic auditory-motor facilitation of gait patterns in patients with Parkinson’s disease. J. Neurol. 62, 22–26. doi: 10.1136/jnnp.62.1.22, PMID: 9010395 PMC486690

[ref45] Morris R. Stuart S. McBarron G. Fino P. C. Mancini M. Curtze C. (2019). Validity of mobility lab (version 2) for gait assessment in young adults, older adults and parkinson’s disease. Physiol. Meas. 40:095003. doi: 10.1088/1361-6579/ab4023, PMID: 31470423 PMC8072263

[ref46] Moussard A. Bigand E. Belleville S. Peretz I. (2014). Learning sung lyrics aids retention in normal ageing and Alzheimer’s disease. Neuropsychol. Rehabil. 24, 894–917. doi: 10.1080/09602011.2014.917982, PMID: 24881953

[ref47] Nieuwboer A. Rochester L. Herman T. Vandenberghe W. Emil G. E. Thomaes T.. (2009). Reliability of the new freezing of gait questionnaire: agreement between patients with Parkinson’s disease and their carers. Gait Posture 30, 459–463. doi: 10.1016/j.gaitpost.2009.07.108, PMID: 19660949

[ref48] Nombela C. (2013). Into the groove: can rhythm influence Parkinson’s disease? Neurosci. Biobehav. Rev. 37, 2, 2564–2570. doi: 10.1016/j.neubiorev.2013.08.003 24012774

[ref49] Patel A. D. Iversen J. R. Chen Y. Repp B. H. (2005). The influence of metricality and modality on synchronization with a beat. Exp. Brain Res. 163, 226–238. doi: 10.1007/s00221-004-2159-8, PMID: 15654589

[ref50] Peterson D. S. Pickett K. A. Duncan R. P. Perlmutter J. S. Earhart G. M. (2014). Brain activity during complex imagined gait tasks in Parkinson disease. Clin. Neurophysiol. 125, 995–1005. doi: 10.1016/j.clinph.2013.10.008, PMID: 24210997 PMC3981914

[ref51] Peterson D. S. Pickett K. A. Duncan R. Perlmutter J. Earhart G. M. (2014). Gait-related brain activity in people with Parkinson disease with freezing of gait. PLoS One 9:e90634. doi: 10.1371/journal.pone.0090634, PMID: 24595265 PMC3940915

[ref52] Pollok B. Krause V. Butz M. Schnitzler A. (2009). Modality specific functional interaction in sensorimotor synchronization. Hum. Brain Mapp. 30, 1783–1790. doi: 10.1002/hbm.20762, PMID: 19301250 PMC6871033

[ref53] Raut R. V. Mitra A. Snyder A. Z. Raichle M. E. (2019). On time delay estimation and sampling error in resting-state fMRI. Neuroimage 194, 211–227. doi: 10.1016/j.neuroimage.2019.03.020, PMID: 30902641 PMC6559238

[ref54] Schlerf J. E. Verstynen T. D. Ivry R. B. Spencer R. M. C. (2010). Evidence of a novel somatopic map in the human neocerebellum during complex actions. J. Neurophysiol. 103, 3330–3336. doi: 10.1152/jn.01117.2009, PMID: 20393055 PMC2888250

[ref55] Sen S. Kawaguchi A. Truong Y. Lewis M. M. Huang X. (2010). Dynamic changes in cerebello-thalamo-cortical motor circuitry during progression of Parkinson’s disease. Neuroscience 166, 712–719. doi: 10.1016/j.neuroscience.2009.12.036, PMID: 20034546 PMC2852615

[ref56] Shulman G. L. Pope D. L. W. Astafiev S. V. McAvoy M. P. Snyder A. Z. Corbetta M. (2010). Right hemisphere dominance during spatial selective attention and target detection occurs outside the dorsal frontoparietal network. J. Neurosci. 30, 3640–3651. doi: 10.1523/JNEUROSCI.4085-09.2010, PMID: 20219998 PMC2872555

[ref57] Taniwaki T. Yoshiura T. Ogata K. Togao O. Yamashita K. Kida H.. (2013). Disrupted connectivity of motor loops in Parkinson’s disease during self-initiated but not externally-triggered movements. Brain Res. 1512, 45–59. doi: 10.1016/j.brainres.2013.03.027, PMID: 23548595

[ref58] Thaut M. H. McIntosh G. C. Hoemberg V. (2014). Neurobiological foundations of neurologic music therapy: rhythmic entrainment and the motor system. Front. Psychol. 5:1185. doi: 10.3389/fpsyg.2014.01185, PMID: 25774137 PMC4344110

[ref59] Thaut M. H. Stephan K. M. Wunderlich G. Schicks W. Tellmann L. Herzog H.. (2009). Distinct cortico-cerebellar activations in rhythmic auditory motor synchronization. Cortex 45, 44–53. doi: 10.1016/j.cortex.2007.09.009, PMID: 19081087

[ref60] Thibault N. Albouy P. Grondin S. (2023). Distinct brain dynamics and networks for processing short and long auditory time intervals. Sci. Rep. 13:22018. doi: 10.1038/s41598-023-49562-8, PMID: 38086944 PMC10716402

[ref61] Vitorio R. Stuart S. Gobbi L. T. B. Rochester L. Alcock L. Pantall A. (2018). Reduced gait variability and enhanced brain activity in older adults with auditory cues: a functional near-infrared spectroscopy study. Neurorehabil. Neural Repair 32, 976–987. doi: 10.1177/1545968318805159, PMID: 30411674

[ref62] Whitehead J. C. Armony J. L. (2018). Singing in the brain: neural representation of music and voice as revealed by fMRI. Hum. Brain Mapp. 39, 4913–4924. doi: 10.1002/hbm.24333, PMID: 30120854 PMC6866591

[ref63] Zarate J. M. (2013). The neural control of singing. Front. Hum. Neurosci. 7:7. doi: 10.3389/fnhum.2013.00237, PMID: 23761746 PMC3669747

